# Mirror neuron system based therapy for aphasia rehabilitation

**DOI:** 10.3389/fpsyg.2015.01665

**Published:** 2015-10-30

**Authors:** Wenli Chen, Qian Ye, Xiangtong Ji, Sicong Zhang, Xi Yang, Qiumin Zhou, Fang Cong, Wei Chen, Xin Zhang, Bing Zhang, Yang Xia, Ti-Fei Yuan, Chunlei Shan

**Affiliations:** ^1^Department of Rehabilitation Medicine, Zhangjiagang Hospital of Traditional Chinese MedicineZhangjiagang, China; ^2^Department of Rehabilitation Medicine, First Affiliated Hospital of Nanjing Medical UniversityNanjing, China; ^3^Department of Rehabilitation Medicine, Zhongda Hospital of Southeast UniversityNanjing, China; ^4^Psychology Department of Shaoxing UniversityShaoxing, China; ^5^The Center for the Study of Language and Cognition of Zhejiang UniversityHangzhou, China; ^6^Department of Radiology, the Affiliated Drum Tower Hospital of Nanjing University Medical SchoolNanjing, China; ^7^School of Psychology, Nanjing Normal UniversityNanjing, China; ^8^Shanghai University of Traditional Chinese MedicineShanghai, China

**Keywords:** mirror neuron system (MNS), action observation treatment (AOT), aphasia, stroke, therapy, rehabilitation, fMRI

## Abstract

**Objective:** To investigate the effect of hand action observation training, i.e., mirror neuron system (MNS) based training, on language function of aphasic patients after stroke. In addition, to reveal the tentative mechanism underlying this effect.

**Methods:** Six aphasic patients after stroke, meeting the criteria, undergo 3 weeks' training protocol (30 min per day, 6 days per week). Among them, four patients accepted an ABA training design, i.e., they implemented Protocol A (hand action observation combined with repetition) in the first and third weeks and carried out Protocol B (static object observation combined with repetition) in the second week. Conversely, for the other two patients, BAB training design was adopted, i.e., patients took Protocol B in the first and third weeks and accepted Protocol A in the second week. Picture naming test, western aphasia battery (WAB) and Token Test were applied to evaluate the changes of language function before and after each week's training. Furthermore, two subjects (one aphasic patient and one healthy volunteer) attended a functional MRI (fMRI) experiment, by which we tried to reveal the mechanism underlying possible language function changes after training.

**Results:** Compared with static object observation and repetition training (Protocol B), hand action observation and repetition training (Protocol A) effectively improved most aspects of the language function in all six patients, as demonstrated in the picture naming test, subtests of oral language and aphasia quotient (AQ) of WAB. In addition, the fMRI experiment showed that Protocol A induced more activations in the MNS of one patient and one healthy control when compared to Protocol B.

**Conclusion:** The mirror neuron based therapy may facilitate the language recovery for aphasic patients and this, to some extent, provides a novel direction of rehabilitation for aphasia patients.

## Introduction

Aphasia is a major disability in patients with stroke. The patients are generally unable to maintain their previous job and suffer from a reduction in social contact. Current aphasia treatments are mostly based on the strategy of “re-education,” by which the speech therapists (or speech-language pathologists, SLP) work intensively with patients to ameliorate or compensate their language impairments. This has been implemented by focusing on instruction and practice of specific language deficits at a behavioral level, which is in contrast to that based on the “brain repair” strategy, by which the disrupted neural circuits are targeted to be activated or repaired at a biological or neurophysiological level (Small, 2009; Small et al., 2013). In this study, we targeted to activate and “repair” a particular neural circuit—mirror neuron system (MNS) by hand action observation training and to verify its effect and mechanism underlying language function recovery in aphasic patients.

Mirror neurons are a specific group of visuomotor neurons, originally discovered in the premotor cortex (area F5) of monkeys. The group of neurons discharge either when the monkey does a particular action or when it observes another individual (monkey or human) doing a similar action (di Pellegrino et al., 1992; Gallese et al., 1996; Rizzolatti et al., 1996). In recent years, a series of brain imaging and neurophysiological studies revealed that a mirror neuron system is also present in the human brain. When an individual observes an action or executes it, a network of cortical areas is activated, including the posterior part of inferior frontal gyrus, the ventral premotor cortex, the inferior parietal lobule and the superior temporal cortex (see Rizzolatti and Craighero, 2004).

Many studies have revealed that MNS play a special role in action imitation, goal-directed action understanding, motor development and motor skill learning (Iacoboni et al., 1999; Nishitani and Hari, 2000; Koski et al., 2002; Buccino et al., 2004; see Rizzolatti et al., 2001; see Rizzolatti and Craighero, 2004). Based on the fact that action observation can lead to MNS activation and plasticity, some researchers adopted action observation treatment (AOT) or MNS activation therapy to accelerate the motor recovery in post-stroke patients (Altschuler et al., 1999; Ertelt et al., 2007; Dohle et al., 2009; Franceschini et al., 2010; Small et al., 2012).

In addition to the effects on motor functions, MNS was also found to be involved in the process of gestural communication, speech and language functions (Fadiga et al., 2002; Tettamanti et al., 2005) and shared by observation and execution of arm or mouth/lip actions and spoken language (Ross et al., 2007; see Gentilucci and Dalla, 2008). It has been shown that patients with Broca's aphasia also show deficits in action understanding and recognition (Fazio et al., 2009). Furthermore, areas of MNS in the left hemisphere, such as the posterior part of the interior frontal gyrus (Broca's area, BA44, 45), inferior parietal lobule (supramarginal and angular gyrus, BA40, 39), and superior temporal cortex (including Wernicke's area, BA20), are all critical language areas. It is reasonable to infer that AOT may result in MNS activation and plasticity (including above mentioned important language areas in left hemisphere) and accordingly facilitate normal language communication in healthy subjects and promote language function recovery in aphasic patients.

AOT for aphasia is currently at an earlier stage on the translational path as compared to motor rehabilitation. Preliminary study (with six aphasic patients) show that observation and execution of action might favor retrieval of action-related words in aphasic patients with a selective deficit for verb retrieval (Marangolo et al., 2010; Bonifazi et al., 2013). Small and his colleagues developed and adopted IMITATE, a computer-assisted system, to train aphasic patients. The data showed that observation of speech action (mouth, lip, and tongue movement) followed by oral repetition of the words and phrases could especially improve repetition for trained stimuli. However, significant differences were not found between control (observe static faces and repeat the words and phrases) and experimental patients and were not found for general language measures pre- and post-therapy as evaluated by Aphasia Quotient (AQ) of western aphasia battery (WAB). The authors admitted in the article that failure of therapeutic effect to generalize to communication skills beyond repetition may indicate theoretical insufficiency or suboptimal implementation (Lee et al., 2010; Duncan et al., 2012).

Therefore, up to now, no studies were found adopting goal-directed hand action observation training for aphasic patients as well as evaluating the effects of this intervention on comprehensive or general language functions (e.g., evaluated by AQ). In particular, no studies were found using neuroimaging techniques such as functional MRI (fMRI) to reveal the mechanism underlying this language function's improvement and its relevance with MNS.

In this study, we applied hand action observation training (Protocol A) and static object observation training as control (Protocol B) to six aphasic patients. WAB, Token Test (for auditory comprehension) and Naming Test using standardized pictures were performed to assess aphasic patients' general and specific language function changes. In addition to the behavioral tests, we also implemented fMRI (which can uncover different activations in MNS between Protocol A and B) to reveal the neural mechanism underlying the language function changes and its relevance with MNS.

Our hypothesis is that hand action observation training may facilitate the MNS activation and plasticity, which is beneficial for language function recovery in aphasic patients.

## Materials and methods

### Participants

Inclusion criteria: First occurrence of a left cerebrovascular accident (CVA) with hematencephalon and cerebral infarction for at least 2 months; aphasia determined by WAB and normal premorbid language function; right-handed according to the Edinburgh Handedness Inventory; greater than 5 years of education (beyond primary school); 35–70 years old; maintenance of partial auditory comprehension ability and tolerance of more than 30 min to complete the daily training; willing to attend our 3 week study.

Exclusion criteria: Intelligence deficit (the normalized score of Raven's Progressive Matrices < 50); obvious articulation, attention, memory, and emotion disturbances; metal implants in the head (unsuitable for fMRI examination); concurrent medical conditions likely to worsen patient's functional status such as: cancer, serious heart, kidney, or liver disease and infectious diseases.

Six aphasic patients conforming to the above inclusion and exclusion criteria were recruited in this study.

#### Case one

The first patient was a 47 year old right-handed male with 15 years of education. He was admitted to the hospital due to right limb movement disorder and loss of consciousness during Feb. 2013. The CT examination revealed the left intracranial hematencephalon, which was then removed with surgery. Following the surgery, the patient recovered with right side movement restriction and speech difficulties. The patient was diagnosed as: (1) Cerebral hemorrhage, right side paralysis, and aphasia; (2) Hypertension, degree III (high risk). He then received 7 months of rehabilitation training including comprehensive training of the hemiplegic limb, occupational therapy, and speech therapy. In Sep. 2013, the follow-up examination on this patient with WAB revealed transcortical motor aphasia (*AQ* = 45.2). Mini-mental state examination (MMSE) score was 18 and Raven's Progressive Matrices (RPM) score was 95, excluding the existence of dementia. Frenchay Dysarthria Assessment revealed no dysarthria.

#### Case two

The second patient was a 39 year old right-handed male with 16 years of education. The patient suffered from sudden right limb paralysis, loss of language function but intact consciousness, lack of nausea/vomiting on Oct. 7, 2013. Head CT examination revealed the left periventricular infarction, and MRI showed acute phase brain infarction spreading in the left frontal lobe, insula, and parietal occipital lobe. The middle and distal segment of the left common carotid artery did not develop in the image, M1 segment stenosis in the left middle cerebral artery as well as intracranial atherosclerosis findings. The patient was diagnosed as: (1) Cerebral infarction with right hemiparesis and aphasia; (2) Hypertension, degree III (very high risk); (3) Type II diabetes. Besides accepting treatments with medicine for controlling hypertension and diabetes, the patient was subjected to 2 months of rehabilitation training including comprehensive training of the hemiplegic limb, occupational therapy and routine speech therapy. On Dec. 3, 2013, the patient came for reexamination with WAB evaluation. The AQ was 41.5 and the aphasia was transcortical mixed aphasia. MMSE score was 14 and Raven's Progressive Matrices (RPM) score was 75, excluding the existence of dementia. Frenchay Dysarthria Assessment revealed no dysarthria.

#### Case three

The third patient was a 55 year old right-handed male, with 8 years of education. He was admitted to the hospital during Oct. 2013 due to acute headache with right limb paralysis. The CT examination revealed hemorrhage in the left basal ganglia, and the patient received a series of treatment to control the intracranial pressure and improve brain functions. After the treatment, the right limb was left with decreased mobility, together with language dysfunction. The patient was diagnosed as: (1) Cerebral hemorrhage, right side paralysis, and aphasia; (2) Hypertension, degree II (high risk). The patient received 1 week of rehabilitation training in the hospital, including comprehensive training of the hemiplegic limb, occupational therapy, and routine speech training. On Dec. 12, 2013, the patient was evaluated with WAB and the AQ was 40.6, showing transcortical motor aphasia. The MMSE score was 9, and the dementia was excluded since his RPM score was 95. Frenchay Dysarthria Assessment revealed no dysarthria.

#### Case four

The fourth patient was a 63 year old right-handed male with 9 years of education. He was admitted to the hospital during Oct. 2011 due to the acute occurrence of language dysfunction, with loss of mobility of the right limbs. The CT revealed acute infarction in the left lateral ventricle, basal ganglia, as well as the frontal (including Broca's area) and temporal lobes. The patient received treatments to prevent platelet aggregation and improve circulation and neuroprotection. After the treatment, the right limb was left with decreased mobility, together with the language dysfunction. The patient was diagnosed as: (1) Cerebral infraction, with right paralysis and aphasia; (2) Secondary epilepsy; (3) Hypertension, degree III (very high risk); (4) Type II diabetes. Afterwards, he received 2 months of rehabilitation training, including comprehensive training of the hemiplegic limb, occupational therapy, and routine speech therapy. On Jan. 3, 2014, the patient was evaluated with WAB and the AQ was 16.2, showing Broca's aphasia. The MMSE score was 10, and the dementia was excluded since his RPM score was 75. Frenchay Dysarthria Assessment revealed no dysarthria.

#### Case five

The fifth patient was a 53 year old right-handed male with 8 years of education. He was admitted to the hospital during Jan. 2013 due to acute right limb paralysis and loss of language function with consciousness intact. The MRI revealed malacia in the left frontal and parietal lobe. The patient was diagnosed as: (1) Cerebral infraction, right paralysis, and aphasia; (2) Hypertension, degree III (very high risk). The patient received rehabilitation training for 4 months, including comprehensive training of the hemiplegic limb, occupational therapy, and routine speech therapy. On Mar. 6, 2014, the patient was evaluated with WAB and the AQ was 55.9, showing transcortical motor aphasia. The MMSE score was 13, and the dementia was excluded since his RPM score was 50. Frenchay Dysarthria Assessment revealed no dysarthria.

#### Case six

The sixth patient was a 45 year old right-handed male with 12 years of education. He was admitted to the hospital during Jun. 2013 due to right limb movement disorder and loss of consciousness. The CT examination revealed hematencephalon in the left basal ganglia. The patient received decompressive craniotomy to control the intracranial pressure and treatment to improve brain functions. After the treatment, decreased mobility on the right limb and language dysfunction were observed. The patient was diagnosed as: (1) Cerebral hemorrhage, (2) Right side paralysis, and aphasia. The patient received 1 month of rehabilitation training in the hospital, including comprehensive training of the hemiplegic limb, occupational therapy and routine speech training. On Jun. 9, 2014, the patient was evaluated with WAB and the AQ was 37.8, showing Broca's aphasia. The MMSE score was 12, and the dementia was excluded since his RPM score was 50. Frenchay Dysarthria Assessment revealed no dysarthria.

All patients' information (cases 1–6) is described in Table 1.

**Table 1:** **General information of all patients**.

**No. of cases**	**1**	**2**	**3**	**4**	**5**	**6**
Gender	Male	Male	Male	Male	Male	Male
Age	47	39	55	63	53	45
Handedness	Right	Right	Right	Right	Right	Right
Job	Manager	Businessman	Worker	Worker	Administrator	Businessman
Education (year)	15	16	8	9	8	12
Course of stroke (month)	7	2	2	26	13	12
Stroke property	Hematencephalon	Cerebral infarction	Hematencephalon	Cerebral infarction	Cerebral infarction	Hematencephalon
Lesion location	Left basal ganglia	Left frontal lobe, insula, parietal and occipital lobe	Left basal ganglia	Left lateral ventricle, basal ganglia, and the frontal and temporal lobes	Left frontal and parietal lobes	Left basal ganglia
Aphasia type	Transcortical motor aphasia	Transcortical mixed aphasia	Transcortical motor aphasia	Broca's aphasia	Transcortical motor aphasia	Broca's aphasia
AQ of WAB	45.2	41.5	40.6	16.2	55.9	37.8
MMSE	18	14	9	10	13	12
RPM	95	75	95	75	50	50

The study has been approved by the ethic committee of medical research concerning humans in Nanjing Medical University. The six patients investigated in this study provided written informed consent. All procedures involved in the present study were in accordance with the guidelines of medical research in Nanjing Medical University.

## Procedure

### Behavioral experiment

#### Training materials and process

In order to investigate whether MNS based treatment approach (e.g., hand action observation) is helpful for aphasic patients, we adopted two types of videos in our study. The first one (experimental materials) contained 140 videos showing different goal- directed *dynamic hand actions* with objects common in daily life, such as cracking a peanut, cutting a water melon, turning on an air conditioner, and etc. The second one (control materials) contained 140 videos showing different *static objects* which are the same as that in aforementioned hand action videos. It is known that steady pictures of different objects lead to no activation of the mirror neuron system (Sahin and Erdogan, 2009).

Accordingly, we used two treatment protocols (Protocol A and B) for the six aphasic patients. In Protocol A (experimental condition), the patients were asked to watch the 80 videos (randomly selected from 140 videos) of hand actions (e.g., cracking a peanut) and hear the name of the objects manipulated (e.g., “peanut”), then repeat them. In Protocol B (control condition), the patients were required to watch the 80 videos of objects (which are the same as the above 80 dynamic videos) and hear the name of the objects (e.g., “peanut”), then repeat them. Therefore, the repetitions in the two protocols are all the same in consideration of excluding the influence of different type of repetition (e.g., repeat “crack a peanut” in Protocol A and repeat “peanut” in Protocol B) on treatment effects.

Each hand action or object video was shown for 7.5 s and repeated 3 times. Therefore, the total time of the 80 videos training was 30 min. Each patient was trained for 6 days per week, with 30 min per day, and 3 weeks in total.

Among the six aphasic patients meeting the inclusion criteria, four patients (the first to the fourth) accepted 3 weeks of ABA training session, i.e., Protocol A was performed in the first and third week and Protocol B was adopted in the second week. ABA design is a common three phase experiment design which can minimize to some extent the bias of results. We also applied BAB training design for the other two patients (the fifth and sixth), i.e., Protocol B was taken in the first and the third week and Protocol A was adopted in the second week, expecting to find more true and consistent effects from the experimental intervention (Protocol A). Please note that the ABA design allows a more reliable establishment of a relationship between intervention and outcome when compared to the AB design. If a change occurs at the onset of the intervention and is reversed at its offset, the two are associated with presence or absence of the intervention, which offers better control for interference of non-interventional factors, e.g., learning/practice effects on the same evaluation materials or fatigue effects after each week's training in this study.

Before and after each training session (i.e., each week), the language function of the patient was evaluated with WAB (4 subtests of oral language and AQ), Token Test and picture naming test. WAB was transferred to Chinese version about 18 years ago (Wang, 1997a,b) and have been adopted in some National projects including National Key Technology Research and Development Program of China during the “10th Five-Year Plan” (Zhang et al., 2004). We adopted WAB not only because the Chinese version of WAB is a very popular test for diagnosis of aphasia in China, but also for its convenience in detecting the abilities of spontaneous speech, auditory comprehension, repetition and naming, as well as providing the general figure of oral language by AQ. The Token Test and picture naming test were supplemented to WAB in order to reveal auditory comprehension and naming abilities in detail.

In the picture naming test, before the training session, the 300 common pictures (with high word frequency familiar to the Chinese) selected from literature (Cycowicz et al., 1997) were randomly presented for 10 s one by one, until the patient failed to name or made mistakes for 60 pictures. Then the 60 pictures were re-tested after the training session (therefore the correct rate before each training session was set to 0). The rest of the pictures were randomly demonstrated and selected again with the same method for the purpose of naming 60 new pictures every week.

The 3 weeks' training were performed by an experimenter. However, the language assessments pre- and post-training were implemented by a speech therapist, who did not know the arrangement of experimental or control condition.

#### Behavioral data statistics

The data was analyzed with SPSS 16.0 (Chicago, US). The accuracy rate (including AQ of WAB, subtests such as spontaneous speech, auditory comprehension, repetition, and naming tests of WAB; Token Test and picture naming test) before and after training were compared (including comparison between pre-training baseline and post- each week's training, and comparison among post- each week's training) by chi-square test (see Tables 2–8). *P* < 0.05 was considered as statistically significant.

**Table 2:** **Picture naming Test**.

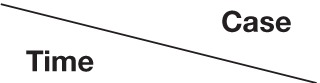	**1**	**2**	**3**	**4**	**5**	**6**
**PICTURE NAMING TEST**						
Before training	0	0	0	0	0	0
First week	47%(28/60)^a^^,^^b^	43%(26/60)^a^^,^^b^	27%(16/60)^a^^,^^b^	22%(13/60)^a^^,^^b^	30%(18/60)^a^^,^^b^	22%(13/60)^a^
Second week	25%(15/60)^a^	23%(14/60)^a^	10%(6/60)^a^	7%(4/60)^a^	56.7%(34/60)^a^	37%(22/60)^a^
Third week	38%(23/60)^a^	47%(28/60)^a^^,^^b^	42%(25/60)^a^^,^^b^	20%(12/60)^a^^,^^b^	36.7%(22/60)^a^^,^^b^	28%(17/60)^a^

a Suggested for P < 0.05 in compared to the results before training;

b Suggested for P < 0.05 in compared to the second week.

**Table 3:** **Spontaneous speech of WAB**.

**Information content**							**Fluency**					
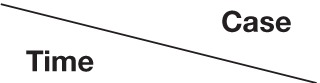	**1**	**2**	**3**	**4**	**5**	**6**	**1**	**2**	**3**	**4**	**5**	**6**
**SPONTANEOUS SPEECH**												
Before training	6/10	3/10	4/10	2/10	5/10	5/10	2/10	4/10	2/10	0/10	4/10	2/10
First week	8/10	5/10	6/10	4/10	5/10	5/10	4/10	4/10	3/10	0/10	4/10	2/10
Second week	5/10	5/10	4/10	2/10	7/10	7/10	3/10	4/10	2/10	0/10	5/10	2/10
Third week	9/10	6/10	7/10	5/10	6/10	5/10	5/10	5/10	3/10	2/10	5/10	3/10

No significant changes were found in spontaneous speech of WAB.

**Table 4:** **Auditory comprehension of WAB**.

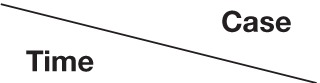	**1**	**2**	**3**	**4**	**5**	**6**
**TOTAL SCORE IN AUDITORY COMPREHENSION**						
Before training	108/200	77/200	104/200	98/200	135/200	96/200
First week	181/200^a^^,^^b^	122/200^a^^,^^b^	170/200^a^^,^^b^	118/200^a^	149/200	112/200^b^
Second week	156/200^a^	99/200^a^	132/200^a^	115/200	157/200^a^	143/200^a^
Third week	189/200^a^^,^^b^	128/200^a^^,^^b^	153/200^a^^,^^b^	152/200^a^^,^^b^^,^^c^	149/200	135/200^a^^,^^c^
**YES/NO ANSWERS**						
Before training	39/60	42/60	39/60	39/60	42/60	45/60
First week	57/60^a^	51/60^a^	54/60^a^	54/60^a^^,^^b^	45/60^b^	42/60
Second week	57/60^a^	45/60	48/60	45/60	54/60^a^	45/60
Third week	60/60^a^	48/60	51/60^a^	57/60^a^^,^^b^	51/60^a^	42/60
**AUDITORY WORD RECOGNITION**						
Before training	45/60	27/60	45/60	43/60	49/60	37/60
First week	58/60^a^	39/60^a^	49/60	41/60	51/60	44/60
Second week	53/60^a^	34/60	40/60	42/60	56/60	50/60^a^
Third week	59/60^a^	41/60^a^	48/60	53/60^a^^,^^b^^,^^c^	53/60	42/60
**SEQUENTIAL COMMANDS**						
Before training	24/80	8/80	20/80	16/80	44/80	14/80
First week	66/80^a^^,^^b^	32/80^a^^,^^b^	67/80^a^^,^^b^^,^^c^	23/80	53/80	26/80^a^^,^^b^
Second week	46/80^a^	20/80^a^	44/80^a^	28/80^a^	47/80	48/80^a^
Third week	70/80^a^^,^^b^	39/80^a^^,^^b^	54/80^a^	42/80^a^^,^^b^^,^^c^	45/80	51/80^a^^,^^c^

a Suggested for P < 0.05 in compared to the results before training;

b Suggested for P < 0.05 in compared to the second week;

c Suggested for P < 0.05 between the first and the third week.

**Table 5:** **Repetition of WAB**.

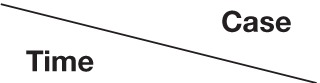	**1**	**2**	**3**	**4**	**5**	**6**
**REPETITION**						
Before training	79/100	74/100	71/100	4/100	86/100	51/100
First week	90/100^a^	88/100^a^^,^^b^^,^^c^	91/100^a^^,^^b^	35/100^a^	86/100^b^	58/100
Second week	88/100	76/100	71/100	44/100^a^	96/100^a^	62/100
Third week	94/100^a^	97/100^a^^,^^b^	87/100^a^^,^^b^	60/100^a^^,^^b^^,^^c^	96/100^a^^,^^c^	60/100

a Suggested for P < 0.05 in compared to the results before training;

b Suggested for P < 0.05 in compared to the second week;

c Suggested for P < 0.05 between the first and the third week.

**Table 6:** **Naming of WAB**.

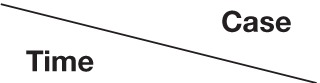	**1**	**2**	**3**	**4**	**5**	**6**
**TOTAL SCORE IN NAMING**						
Before training	13/100	25/100	20/100	8/100	36/100	20/100
First week	54/100^a^^,^^b^	54/100^a^^,^^b^	26/100	9/100	51/100^a^^,^^b^	26/100
Second week	33/100^a^	40/100^a^	17/100	13/100	68/100^a^	35/100^a^
Third week	46/100^a^^,^^b^	62/100^a^^,^^b^	36/100^a^^,^^b^	29/100^a^^,^^b^^,^^c^	62/100^a^	31/100
**OBJECT NAMING**						
Before training	9/60	20/60	15/60	8/60	30/60	17/60
First week	40/60^a^^,^^b^	45/60^a^^,^^b^	22/60	9/60	38/60^10^	21/60
Second week	21/60^a^	34/60^a^	14/60	9/60	52/60^a^	28/60^a^
Third week	38/60^a^^,^^b^	47/60^a^^,^^b^	29/60^a^^,^^b^	27/60^a^^,^^b^^,^^c^	45/60^a^	25/60
**WORD FLUENCY**						
Before training	0/20	1/20	0/20	0/20	0/20	1/20
First week	2/20	1/20	0/20	0/20	1/20	3/20
Second week	4/20	2/20	0/20	0/20	0/20	2/20
Third week	4/20	5/20	0/20	0/20	1/20	4/20
**SENTENCE COMPLETION**						
Before training	2/10	2/10	2/10	0/10	4/10	0/10
First week	6/10	6/10	2/10	0/10	8/10	0/10
Second week	6/10	2/10	1/10	2/10	8/10	0/10
Third week	8/10	4/10	4/10	0/10	10/10^a^	1/10
**RESPONSIVE SPEECH**						
Before training	2/10	2/10	3/10	0/10	2/10	2/10
First week	6/10	2/10	2/10	0/10	4/10	2/10
Second week	2/10	2/10	2/10	2/10	8/10^a^	5/10
Third week	6/10	6/10	3/10	2/10	6/10	1/10

a Suggested for P < 0.05 in compared to the results before training;

b Suggested for P < 0.05 in compared to the second week;

c Suggested for P < 0.05 between the first and the third week.

**Table 7:** **Aphasia quotient of WAB**.

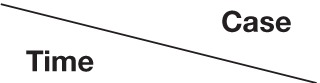	**1**	**2**	**3**	**4**	**5**	**6**
**APHASIA QUOTIENT (AQ)**						
Before training	45.2/100	41.5/100	40.6/100	16.2/100	55.9/100	37.8/100
First week	70.9/100^a^^,^^b^	58.6/100^a^	58.4/100^a^^,^^b^	28.6/100^a^	60.3/100^b^	42/100
Second week	55.8/100^a^	51.1/100	42.8/100	26.9/100	73.5/100^a^	51.7/100^a^
Third week	76.9/100^a^^,^^b^	66.6/100^a^^,^^b^	59.9/100^a^^,^^b^	47/100^a^^,^^b^^,^^c^	68.5/100	47.7/100

a Suggested for P < 0.05 in compared to the results before training;

b Suggested for P < 0.05 in compared to the second week;

c Suggested for P < 0.05 between the first and the third week.

**Table 8:** **Token score of WAB**.

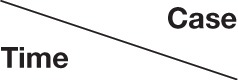	**1**	**2**	**3**	**4**	**5**	**6**
**TOKEN SCORE**						
Before training	8/36	3/36	9/36	5.5/36	11/36	7/36
First week	11/36	6/36	8.5/36	5/36	11.5/36	6/36
Second week	8.5/36	5/36	8/36	4/36	14/36	5/36
Third week	13/36	6/36	9.5/36	5.5/36	12/36	6/36

No significant changes were found in spontaneous speech of WAB.

### fMRI experiment

#### Participants and fMRI paradigm design

An aphasic patient (Case two in the behavioral experiment) and one healthy subject (24 years old), both right-handed males, were included in the fMRI experiment. They signed the informed consent for fMRI and all procedures were under the ethical guidelines.

**Figure 1:**
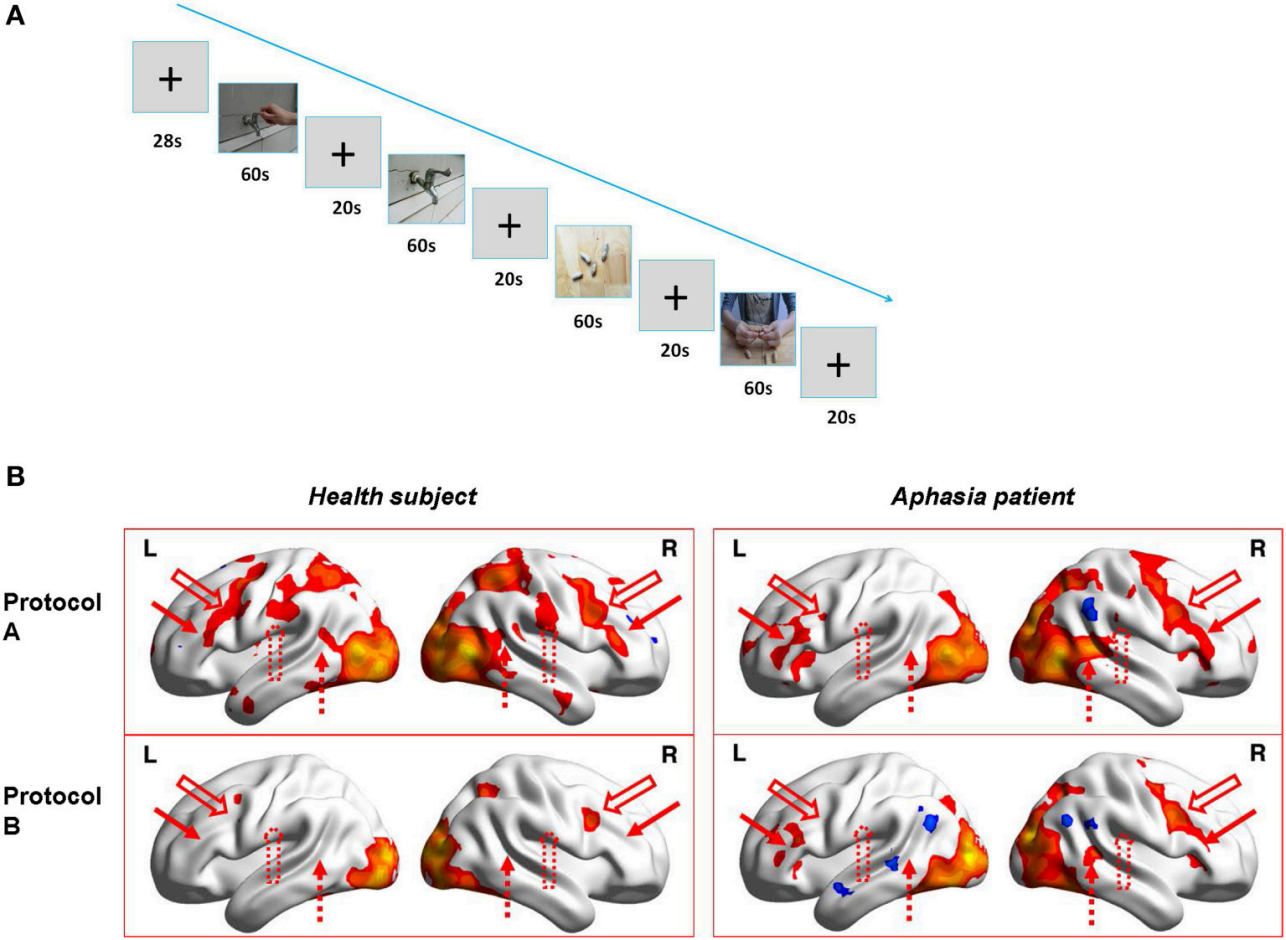
**The fMRI experiment design and results. (A)** Figure Demo of the video blocks of Run1 in fMRI experiment (i.e., Protocol ABBA; Run2 is Protocol BAAB). **(B)** 3D illumination of activations for the healthy subject (left) and the patient (right) by Protocol A (upper) and Protocol B (lower). L and R indicate Left and right hemisphere, respectively. Upper left: healthy subject with protocol A; Upper right: patient with protocol A; Lower left: healthy subject with protocol B; Lower right: Patient with protocol B. Red solid arrow is corresponding to posterior inferior frontal gyrus (BA 44-45, left is Broca's area), red hollow arrow is ventral premotor cortex (BA6), red dotted hollow arrow is supramarginal gyrus(BA40), and red dotted arrow indicates posterior superior temporal gyrus (BA 22, left is Wernicke's area).

During fMRI scanning, the two subjects were presented alternatively two runs of blocks of hand action video (Protocol A) and static object video (Protocol B) (Run1 is ABBA, Run 2 is BAAB, see Figure 1A). Each block of the video lasted 60 s (20 video fragments, each for 3 s) and the rest of the blocks (see crosshair “+”) lasted 20 s (the first block is 28 s and the 8 s images were discarded to confirm the stability of MRI signals.). Therefore, the total time of each run was 348 s.

In order to verify that the subjects watched the videos continuously and were not distracted, they were asked to press a button on the response box as soon as they saw the video of basketball playing (20% in Protocol A) or a basketball on the table (20% in Protocol B).

#### MRI data acquisition

The studies were performed on a Philips Achieva 3.0 T TX dual Medical Systems using an 8-channel phased array coil. MR images of the entire brain were acquired using echo planar imaging (EPI) with the following parameters: *TR* = 2000 ms, *TE* = 30 ms, Flip Angle = 90°, FOV = 192^*^192^*^140 mm^3^, Acquisition Matrix = 64^*^64, number of slices = 35, slice thickness = 4 mm and number of repetitions = 180. A 3D T1-weighted, high-resolution anatomical image set was also acquired from each subject for functional map overlay with the following parameters: *TR* = 9.8 ms, *TE* = 4.6 ms, Flip Angle = 8°, FOV = 200^*^200^*^192 mm^3^, Acquisition Matrix = 200^*^180, number of slices = 192, slice thickness = 1 mm.

#### fMRI processing and analysis

The fMRI images were re-aligned, co-registered, and normalized to the Montreal Neurological Institute brain template using SPM8 [http://www.fil.ion.ucl.ac.uk/spm/]. The fMRI images were normalized, re-sliced into 3^*^3^*^3 mm^3^ voxels before applying a smoothing Gaussian kernel of 8^*^8^*^8 mm^3^ (full width at half maximum). Activation maps for each subject were computed using general linear models (GLMs), including regressors representing the hand action and the static object observation conditions. The contrast between these conditions was used to produce statistic parametric maps for individual subjects (*P* < 0.05, Alphasim correction, Number of Clusters > 389).

## Results

### The accuracy rate of picture naming test

**Figure 2:**
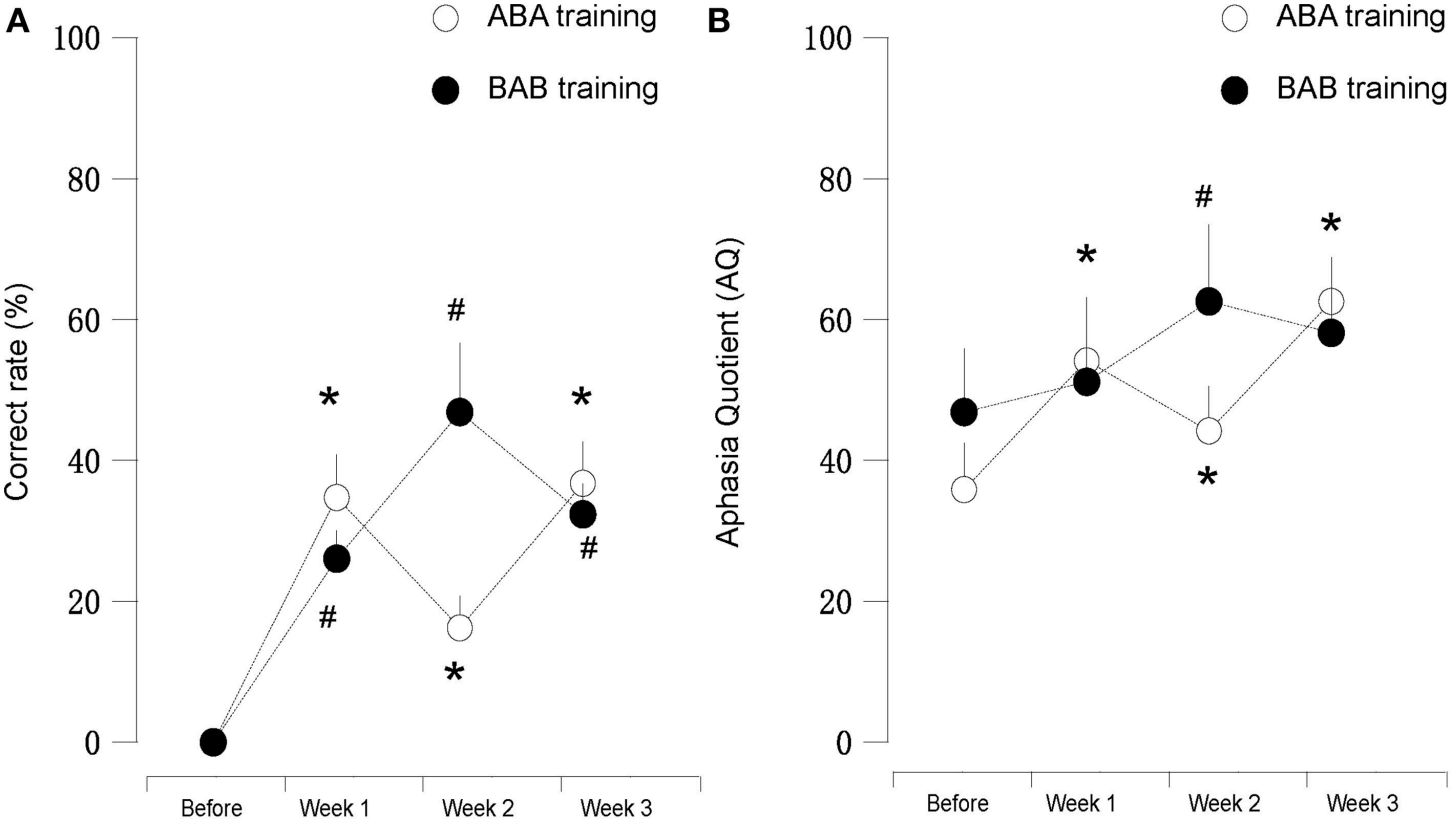
**(A)** (left): Correct rate and **(B)** (right): AQ score of patients subjected to mirror neuron system activation training. “A” or “B” in the panel indicated different protocols employed in this study. We found that the results in first (Protocol A) and third weeks (Protocol A) were clearly better than the second week (Protocol B). Patients five and six (black dot, Protocol B-A-B) showed vice versa. ^*^ and # suggested for *P* < 0.05 in compared to the before training stage.

We found that during the “ABA” training session the accuracy rate of picture naming in the four patients steadily increased for 3 weeks, in comparison to the performance before the training. Interestingly, the accuracy rate decreased at the second week (Protocol B), in comparison to the first week (Protocol A). Then the accuracy rate increased again with Protocol A in the third week, in comparison to the second week (Protocol B), i.e., the results appear as a “V” curve (Figure 2).

Interestingly, with “BAB” design training, the patients five and six demonstrated exactly the opposite. The accuracy rate increased significantly in the second week (Protocol A), and decreased again in the third week (Protocol B), i.e., the results appear as an inverted “V” curve (Figure 2).

### The WAB and token scores with the training

We found that the majority of the WAB subtests and AQ demonstrated similar pattern of changes as the accuracy rate in the picture naming test in all six patients during this 3 week training. The Token score did not demonstrate such trends.

All the results of language function tests and statistical analysis (comparison between 4 evaluation time points) are shown in Tables 2–8 and Figure 2.

### fMRI results and comparison of fMRI activations between protocol A and B

The sequence of the fMRI experiment is shown in Figure 1A. The results (Figure 1B) indicate that for the healthy subject, (left) Protocol A (upper) induced wide activations in bilateral hemispheres which included premotor cortex (BA6), ventral post-central gyrus (BA1-3), posterior inferior frontal gyrus (BA44-45, left is Broca's area), superior parietal lobule (BA5,7), supramarginal gyrus (BA40), posterior superior temporal gyrus (BA22, left is Wernicke's area), occipital cortex (BA17-19), ventral occipiotemporal cortex(BA37), and temporal pole (BA38), whereas Protocol B (lower) only activated bilateral occipital cortex (BA17-19), tiny premotor cortex(BA6) and right superior parietal lobule(BA7). Therefore, MNS (Broca's area and its homolog in right hemisphere, ventral premotor cortex, supramarginal gyrus and Wernicke's area) were probably only activated in Protocol A (hand action observation) and not in Protocol B (static object observation). However, as for the patient, (right) it demonstrated different results. The activations induced by Protocol A (upper) in the right hemisphere were similar (except activations in supramarginal gyrus) to those of the healthy subject, but not in left hemisphere. There were only small area activations in Broca's area (BA44-45), ventral premotor cortex (BA6) as well as occipital cortex (BA17-19) and ventral occipiotemporal cortex (BA37). Compared to Protocol A, Protocol B (lower) induced less activations in left Broca's area (BA44-45) and no activation in left ventral premotor cortex (BA6). In addition, Protocol B (lower) also resulted in less activations in the right hemisphere, including right superior premotor cortex(BA6), posterior inferior frontal gyrus(BA44-45), superior parietal lobule(BA5), posterior superior temporal gyrus(BA22), and no activations in the right ventral post-central gyrus(BA 1-3) and supramarginal gyrus(BA40).

## Discussion

MNS is an action observation and execution matching system, which can be activated by action observation. It is also a neural network shared by motor and language processing (Ross et al., 2007; see Gentilucci and Dalla, 2008). It has been shown that the activation of MNS to be of great significance in motor and language function development in healthy subjects (Nishitani and Hari, 2000; Koski et al., 2002; Buccino et al., 2004; see Rizzolatti et al., 2001; see Fadiga et al., 2002; Rizzolatti and Craighero, 2004; Tettamanti et al., 2005).

Although there have been several studies adopting action observation treatment (AOT) or MNS activation therapy to accelerate motor recovery in post-stroke patients (Altschuler et al., 1999; Ertelt et al., 2007; Dohle et al., 2009; Franceschini et al., 2010; Small et al., 2012), there are few studies reporting the effects of AOT on language recovery for aphasic patients.

AOT for aphasia is currently at a developing period. Preliminary studies (with 6 aphasic patients) showed that observation and execution of action might favor retrieval of action-related words in aphasic patients (Marangolo et al., 2010; Bonifazi et al., 2013). Duncan and his colleagues reported that observation of speech action (mouth, lip and tongue movement) followed by oral repetition of the words and phrases could improve repetition rather than general language function evaluated by AQ of WAB (Duncan et al., 2012).

As far as we know, no studies adopting hand action observation training for aphasic patients and evaluating its effects on comprehensive or general language functions (e.g., evaluated by AQ). Especially, no reports were found using neuroimaging techniques such as fMRI to reveal the mechanism underlying language functions improvement by AOT and its relevance with MNS.

The purpose of this preliminary study is to verify our hypothesis, i.e., hand action observation training may facilitate the MNS activation and plasticity, which is beneficial for language function recovery in aphasic patients.

We explore the efficacy of hand action observation combined with repetition of object names on the general and specific language functions in six aphasic patients. To our knowledge, this is the first study investigating the usefulness of hand action observation combined with repetition on general language functions measured by AQ of WAB. Meanwhile, we applied fMRI to reveal the possible neural mechanism underlying language function improvement by hand action observation and its relationship with MNS activation.

In our study, Protocol A (hand action observation associated with repetition), which could activate MNS, demonstrated its obvious efficacy on language function improvement evaluated by WAB and naming test. On the other hand, Protocol B (static object observation associated with repetition), which did not initiate MNS activation (Sahin and Erdogan, 2009), demonstrated decreased effects on language deficits amelioration, in comparison to Protocol A. The distinctive effects on naming test and AQ between Protocol A and B were shown in Figure 2 (like a “V” curve indicating the high-low-high response with ABA sequential training and inverted “V” curve marking the low-high-low response in BAB sequential training during 3 weeks' training) and Tables 2–8.

Furthermore, Tables 2–8 indicated that Protocol A had relatively general and robust effects on language impairment recovery. The effect of Protocol A is not only for transcortical (motor or mixed) aphasia (patients 1–3 and 5) but also for perisylvian (Broca's) aphasia (patients 4 and 6). In addition, Protocol A is not only fit for patients with lesions in subcortical area (patients 1, 3, and 6) but also for patients with lesions in cortical areas (patients 2 and 5) or with combining areas (patients 4). Furthermore, Protocol A is not only effective for patients with stroke within 3 months (patients 2 and 3), but also for patients with stroke more than 6 months (patient 1), 1 year (patients 5 and 6) or even 2 years (patients 4).

Token Test results showed no significant improvement before and after Protocol A and B treatment. This may result from the fact that comparing to WAB, the Token Test not only checks the patient's auditory comprehension, but also the recognition of Token items with different color, shape, and size, which need normal manipulation ability with the hand to point to corresponding items (all six patients had right hemiplegia). Furthermore, the total score of the Token Test is only 36, there may also be statistical disadvantages in showing significant changes after short term (3 weeks) training.

Since the duration, intensity, frequency, total time of training as well as word repetition are all the same between Protocol A and B, the only difference between the two protocols is whether there is hand action (Protocol A) or static object observation (Protocol B), We assumed that the effects of Protocol A on language improvement was mainly derived from MNS activation and plasticity (including critical language areas) through 3 weeks of hand action observation training.

In order to confirm our assumption, we implemented preliminary fMRI experiments in a patient and a healthy subject. The results of the fMRI experiment showed that Protocol A induced activations in the healthy subject's mirror neuron system (MNS), including bilateral posterior inferior frontal gyrus (BA 44-45, left is Broca's area), ventral premotor cortex (BA6), supramarginal gyrus (BA40), and posterior superior temporal gyrus (BA 22, left is Wernicke's area) while Protocol B did not. For the patient, Protocol A induced more activations in MNS (bilateral BA 44-45, BA 6, right BA 40, BA22) when compared to Protocol B. However, the left supramarginal gyrus (BA40) and Wernicke's area (BA22) were not activated during both protocols, which may be related to the fact that there were wide lesions in the aphasic patient's left hemisphere. In this fMRI experiment, we applied enough stimulus (2 runs and 8 blocks), counterbalanced design (Run1 is ABBA, Run 2 is BAAB) to avoid possible bias or weakness. We also required the subjects to perform specific tasks (press a button in the response box as soon as they saw the video of playing basketball or a basketball on the table in Protocol A or B, respectively) during fMRI scan, which ensured the subjects' compliance to the requirements of experiment. Therefore, the fMRI findings may provide us preliminary evidence supporting our assumption, although the number of subjects was limited.

To our knowledge, this is the first study reporting that hand action observation training could facilitate the improvement of general language function for aphasic patients. Although Duncan et al. also adopted AQ to indicate general language recovery before and after treatment, they did not find significant improvement in AQ after speech action (mouth, lip, and tongue movement) observation training (Duncan et al., 2012). This may imply that hand action observation training is a better approach than speech/mouth action observation training for promoting general or comprehensive language function recovery for aphasic patients.

As we introduced in the *Training Materials and Process* Section, ABA and BAB design in our study (*N* = 6) may reduce, to some extent, the interference from non-training factors such as learning/practice effects or fatigue effects. For example, in ABA design, the improvement of language function after the first week of training (Protocol A) should not result from learning/practice effects (using the same WAB test before and after the first week) when considering the decreased effect after the second week of training (Protocol B). Similarly, the decreased effect after the second week of training should not be induced from fatigue effects when considering the rebounded effect after the third week of training (Protocol A). It is the same for the BAB design.

Despite taking relatively reasonable consideration for our behavioral and fMRI experiments, there are limitations in this study. First, the number of the participant is limited (6 for behavioral and 2 for fMRI experiment). Second, the duration of the study is also limited (3 weeks). Third, the patients were not randomly assigned to ABA or BAB sessions.

Therefore, the findings from our behavioral and fMRI experiments provide preliminary evidence or tendency supporting our hypothesis that MNS based hand action observation training may facilitate the MNS activation and plasticity. This is beneficial for language function recovery in aphasic patients.

We should recruit more aphasic patients in the future and perform randomized control trials with longer duration to integrate better behavioral and functional neuroimaging approaches that may provide more persuasive results.

## Author contributions

WC, QY, XJ, SZ, YX, TY, and CS designed the study; WC, QY, XJ, SZ, XY, QZ, FC, WC, XZ, and BZ performed the experiment; all authors analyzed the data together and wrote the paper together.

### Conflict of interest statement

The authors declare that the research was conducted in the absence of any commercial or financial relationships that could be construed as a potential conflict of interest.
